# How we manage patients with retinoblastoma

**Published:** 2018-06-03

**Authors:** Vikas Khetan

**Affiliations:** 1 Senior Consultant: Sankara Nethralaya, Chennai, India.

When a child with retinoblastoma reports to our centre, a message is immediately passed on to a physician who treats retinoblastoma.

The child is then expedited to reach the physician, where a proper history is taken. After initial evaluation, drops are applied for pupillary dilatation. After the dilatation, the fundi are examined and a quick assessment of tumor volume and initial staging and grouping of the tumour in the eyes is made.

The child then undergoes ultrasound of both eyes, irrespective of it being unilateral presentation. An MRI of the orbits and brain is then advised. The MRI usually happens the same day and reporting takes place within a few hours. Once this information is available, the child is scheduled for an examination under general anaesthesia. Following this, the treatment plan is discussed with the parents.

**“If enucleation is planned, we always ask for an opinion from another retinoblastoma expert.”**

In case of an orbital presentation, or an MRI showing optic nerve involvement, additional testing in the form of cerebrospinal fluid (CSF) analysis and a bone marrow aspirate is conducted and evaluated. Staging of the tumour is then performed as per the tests.

If enucleation is planned, we always ask for an opinion from another retinoblastoma expert.

The option of performing genetic testing is also discussed with the parents; however, this is not routinely done as a standard of care as the testing is often expensive and not many parents can afford it. Our aim therefore shifts to the management of the child.

We have an ocular oncologist in the team who visits our hospital to examine these children in case they require chemotherapy. We are also equipped to perform brachytherapy when needed.

